# bFGF-Mediated Inhibition of Astrocytes’ Optogenetic Activation Impairs Neuronal Repair in Female Rats After Stroke

**DOI:** 10.3390/ijms26136521

**Published:** 2025-07-07

**Authors:** Xinfa Shao, Yangqianbo Yao, Victoria Shi, Qian Suo, Shengju Wu, Han Wang, Muyassar Mamtilahun, Wanlu Li, Yaohui Tang, Guo-Yuan Yang, Qun Xu, Zhijun Zhang

**Affiliations:** 1 Shanghai Jiao Tong Affiliated Sixth People’s Hospital, School of Biomedical Engineering, Shanghai Jiao Tong University, Shanghai 200030, China; dimefluthrin@sjtu.edu.cn (X.S.); sensiblebobo@sjtu.edu.cn (Y.Y.); victoria.shi@gmx.de (V.S.); suoqian@sjtu.edu.cn (Q.S.); wsj1548491481@163.com (S.W.); 123082910026@sjtu.edu.cn (H.W.); muyassar0729@163.com (M.M.); liwanlu-0424@sjtu.edu.cn (W.L.); yaohuitang@163.com (Y.T.); gyyang0626@163.com (G.-Y.Y.); 2 Department of Health Management Center, Ren Ji Hospital, Shanghai Jiao Tong University School of Medicine, Shanghai 200127, China

**Keywords:** astrocyte, bFGF, gender, optogenetics, stroke

## Abstract

Astrocyte activation and gender differences play critical roles in the prognosis following stroke. Recent studies have shown that optogenetic technology can promote brain repair after stroke by activating astrocytes in male rats. However, it remains unclear whether gender differences influence the efficacy of optogenetic activation of astrocytes in regulating post-stroke brain repair and its underlying mechanisms. In this study, we activated astrocytes in the ipsilateral cortex of adult glial fibrillary acidic protein-channelrhodopsin 2-enhanced yellow fluorescent protein (GFAP-ChR2-EYFP) transgenic Sprague Dawley rats using optogenetic stimulation at 24, 36, 48, and 60 h after inducing photothrombosis stroke. Neurobehavioral tests, cresyl violet staining, RT-qPCR, Western blot, and immunofluorescence analysis were performed on both female and male rats. Our results showed that male rats exhibited significant improvements in behavioral scores and reduction in infarct size after optogenetic activation of astrocytes at three days post-stroke (*p* < 0.05), whereas no significant changes were observed in female rats. Additionally, in female rats, the expression of basic fibroblast growth factor (bFGF) increased after ischemic stroke and astrocytic optogenetic stimulation (*p* < 0.05), leading to enhanced endothelial cell proliferation compared to male rats (*p* < 0.05). In vitro experiments further demonstrated that the astrocyte activation was inhibited in the presence of bFGF (*p* < 0.05). These findings suggest that the increase in bFGF levels in females following stroke may inhibit the optogenetic activation of astrocytes, thereby attenuating the therapeutic effect of astrocyte activation on post-stroke brain repair. This study provides important insights into the gender-specific roles of astrocytes in the acute phase of ischemic stroke.

## 1. Introduction

Astrocytes play a pivotal role in ischemic stroke, where they not only passively support the neuronal survival and development but also actively regulate synaptic transmission and integrity of blood–brain barrier (BBB) [1,2,3,4]. Astrocytes become activated following ischemic stroke, exhibiting both detrimental and protective effects. The harmful effects are primarily mediated through the release of inflammatory factors such as interleukin-6 (IL-6), tumor necrosis factor-α (TNF-α), interleukin-1α (IL-1α), interleukin-1β (IL-1β), interferon γ (IFNγ), and inducible nitric oxide synthase (iNOS), while the protective effects are linked to the release of anti-inflammatory factors, including interleukin-10 (IL-10), interleukin-33 (IL-33), matrix metalloproteinase-2 (MMP2), matrix metalloproteinase-9 (MMP9), vascular endothelial growth factor (VEGF), and bFGF [5,6,7,8,9]. Meanwhile, studies have shown that astrocytes have two activated states under pathological conditions, which are termed A1 and A2 astrocytes [10]. A1 astrocytes exert detrimental effects, while A2 astrocytes contribute to neuro-protection [10]. Understanding the functional transformation of astrocytes after ischemic stroke is crucial for the development of effective treatments.

Optogenetics is a powerful technique that enables precise control of cellular activity, with high specificity and temporal precision [11,12,13]. Astrocytes expressing channelrhodopsin 2 (ChR2) can be activated by blue light, allowing for the study of their role in neural communication and brain function regulation [14,15]. Recent evidence suggests that optogenetically manipulated astrocytes affect brain function by releasing gliotransmitters [16,17,18]. Light-induced activation of astrocytes expressing ChR2 increases calcium influx, which triggers the release of cytokines and gliotransmitters, modulating the activity of neighboring neurons [19]. Additionally, astrocytes help maintain BBB function by interacting closely with endothelial cells and covering microvessels [20]. These findings highlight the potential impact of astrocyte activation on endothelial cells, particularly after ischemic stroke.

Basic fibroblast growth factor (bFGF) is a key regulator of fibroblasts’ function, promoting their growth, differentiation, and regeneration. It has broad applications in disease treatment, particularly for its role in the nervous system [21]. bFGF supports angiogenesis, mediates astrocyte activation, and promotes astrocyte proliferation and differentiation [22,23,24,25]. In the central nervous system, bFGF is abundant and has potent neurotrophic effects, supporting the survival and growth of neurons, astrocytes, and endothelial cells both in vitro and in vivo [26].

Sex hormones significantly influence both the onset and prognosis of stroke. The risk of stroke varies by sex: men face a higher risk at a young age, while women experience a significantly increased risk after menopause [27]. These differences may be attributed to the distinct effects of sex hormones on vascular and neuronal function [28]. Moreover, sex hormones can affect stroke pathogenesis, with estrogen generally exerting a protective effect [29]. After ischemic stroke, astrocytes can sense the steroid levels and produce steroid hormones themselves, contributing to neural tissue response to inflammation and injury [30]. This study explores the gender differences in astrocytes’ response to ischemic stroke by examining the expression of neurotrophic and inflammatory factors, as well as the underlying mechanisms involved.

## 2. Results

### 2.1. Astrocytic Optogenetic Stimulation Has No Effect on Neurobehavioral Outcomes in Female Rats During Acute Phase of Stroke

**Figure 1:**
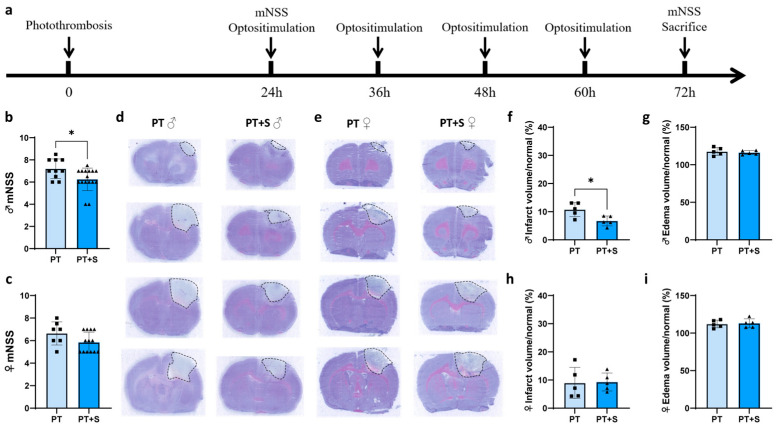
Astrocytic photogenetic stimulation has no effect on neurobehavioral outcomes in female rats after stroke. (**a**) Animal experiment design in vivo. (**b**,**c**) mNSS of male rats (**b**) and female rats (**c**) on day 3 after stroke. (Square: PT group. Triangle: PT + S group. N = 10 rats in male PT group. N = 16 rats in male PT + S group. N = 7 rats in female PT group. N = 13 rats in female PT + S group, Mann–Whitney U test. * *p* < 0.05.) (**d**,**e**) Representative images of cresyl violet staining of male rats (**d**) and female rats (**e**) on day 3 after stroke. Dashed lines indicate cerebral infarction. (**f**–**i**) Quantification of the percentage of brain infarct volume ((**f**) male rats and (**h**) female rats) and edema volume ((**g**) male rats and (**i**) female rats) on day 3 after stroke. (Square: PT group. Triangle: PT + S group. N = 5 rats in male PT group. N = 5 rats in male PT + S group. N = 5 rats in male PT group. N = 5 rats in male PT + S group, *t* test. * *p* < 0.05.) PT: photothrombosis. PT + S: photothrombosis with laser stimulation.

**Figure A1:**
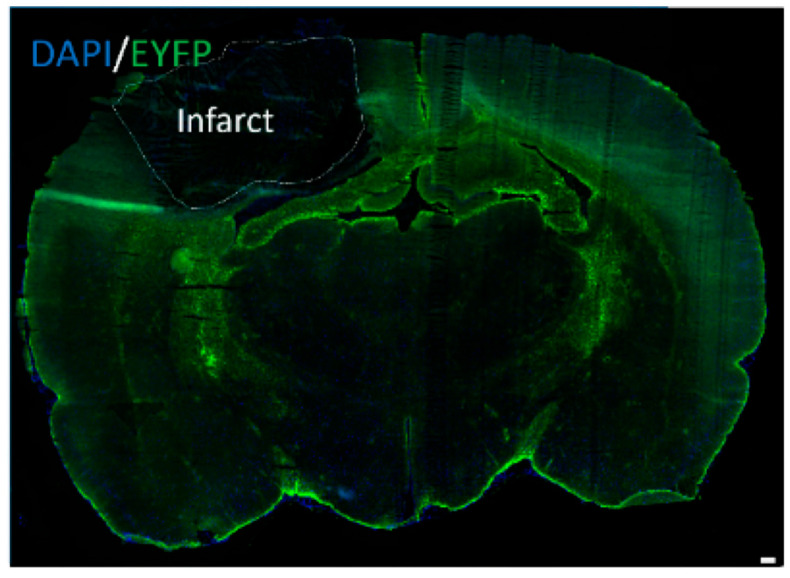
Optogenetic stimulation successfully activates astrocytes in rats. Image of large picture splicing in PT + S group. DAPI shows the overall brain area and EYFP counts for optogenetics-activated astrocytes. The infarct area is presented as a dotted line. PT + S: photothrombosis with laser stimulation. Bar = 50 μm.

We first validated the external laser stimulation method using immunofluorescent staining (Figure A1). The experimental design is shown in Figure 1a. We then assessed neurological function using the modified neurologic severity score(mNSS) 3 days after stroke. The data showed significant improvement in the photothrombotic stroke group with laser stimulation (PT + S) in male rats compared to the photothrombotic stroke group without laser stimulation (PT). However, no significance differences were observed in female rats (Figure 1b,c), indicating that male rats showed better neurological recovery following astrocytic photogenetic stimulation during acute phase of stroke.

To further investigate the impact of astrocyte optogenetic activation on brain tissue repair, we performed cresyl violet staining of brain slices to assess infarct volume and edema on day 3 after PT stroke. The results showed that infarct volume decreased in PT + S compared to PT rats of the male group, and edema volume had no differences in PT + S compared to PT rats of both groups (Figure 1d–i).

### 2.2. bFGF Expression Upregulated in Female Photothrombotic Rats After Astrocytic Photogenetic Stimulation

**Figure 2:**
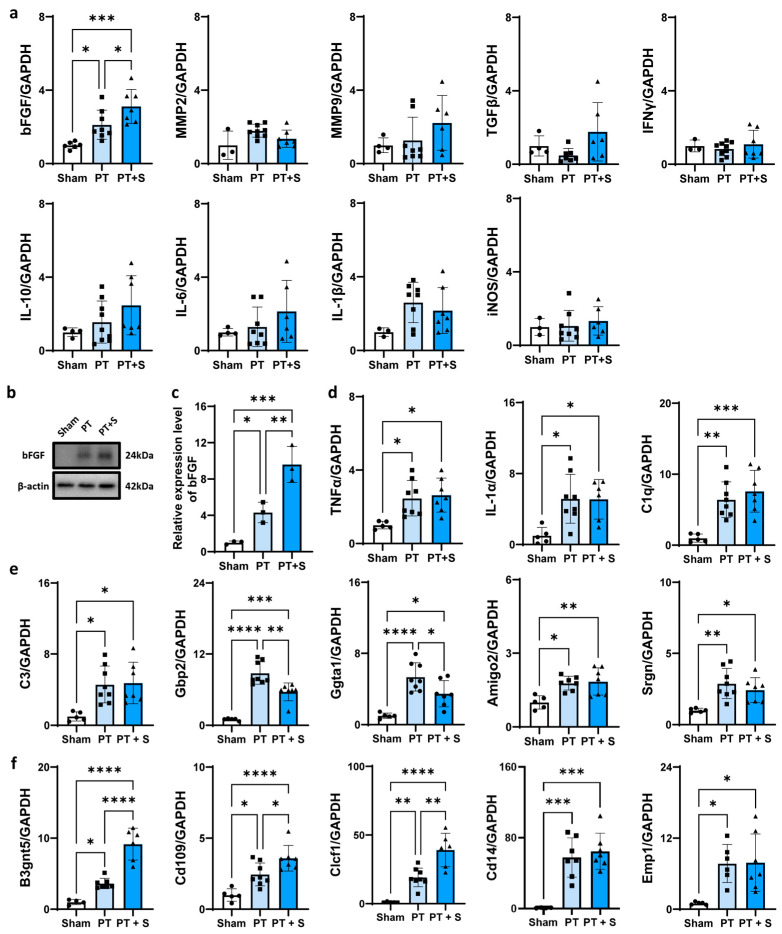
The expression of bFGF upregulated in female photothrombosis rats. (**a**) The mRNA expression level of bFGF, MMP2, MMP9, TGFβ, IFNγ, IL-10, IL-6, IL-1β, and iNOS in different groups. N = 3~6 for Sham group. N = 6~9 for PT group. N = 6~7 for PT + S group. (**b**) Western blot analysis for the expression level of Hi-bFGF in different groups. N = 3 for each group. (**c**) Quantification of Hi-bFGF relative to β-actin and normalized to sham group. N = 3 for each group. (**d**) The mRNA expression level of microglia-secreted astrocyte-activating factors TNFα, IL-1α, and C1q in different groups. N = 5 for Sham group. N = 8 for PT group. N = 6~7 for PT + S group. (**e**) The mRNA expression level of A1-specific transcripts complement component 3 (C3), Gbp2, Ggta1, adhesion molecule with Ig-like domain 2 (Amigo2), and serglycin (Srgn) in different groups. N = 5 for Sham group. N = 7~8 for PT group. N = 7 for PT + S group. (**f**) The mRNA expression level of A2-specific transcripts B3gnt5, Cd109, Clcf1, cluster of differentiation 14 (Cd14), and epithelial membrane protein 1 (Emp1) in different groups. N = 4~5 for Sham group. N = 6~8 for PT group. N = 6~7 for PT + S group. Circle: Sham group. Square: PT group. Triangle: PT + S group. All data are presented as mean ± SD. One-way ANOVA test. * *p* < 0.05, ** *p* < 0.01, *** *p* < 0.001, and **** *p* < 0.0001.

To explore the potential mechanism underlying the sex-specific differences in response to astrocytic photogenetic stimulation, we analyzed the expression of inflammatory factors in three experimental groups. We found that bFGF expression significantly increased in female rats in both the PT and PT + S groups compared to controls, while there is no significant increase in bFGF in male rats in our previous research [31]. In contrast, there were no significant changes in the expression of other inflammatory markers, such as IL-10, IL-6, IL-1β, transforming growth factor-β (TGFβ), IFNγ, iNOS, MMP2, and MMP9 (Figure 2a). This suggested that bFGF upregulation in female rats may be a key factor contributing to sex differences after astrocytic optogenetic stimulation. Western blot analysis further confirmed the increased expression of bFGF in the PT + S group compared to the PT group (Figure 2b,c). Additionally, we assessed the activation state of astrocytes. A1 astrocytes can be induced by microglia-derived factors, including TNF-α, IL-1α, and complement component 1q (C1q) [10]. RT-PCR results showed that TNF-α, IL-1α, and C1q were significantly upregulated in the PT group compared to the sham group, while no significant changes were observed between the PT + S group and PT group (Figure 2d). Meanwhile, guanine-binding protein 2 (Gbp2) and α-1,3-galactosyltransferase (Ggta1) (A1-specific transcripts) were significantly downregulated after laser stimulation, while β-1,3-N-acetylglucosaminyltransferase 5 (B3gnt5), cluster of differentiation 109 (Cd109), and cardiotrophin-like cytokine factor 1 (Clcf1) (A2-specific transcripts) showed a significant increase (Figure 2e,f). These results suggested that astrocyte optogenetic activation facilitates the A2 phenotype.

### 2.3. Upregulation of bFGF Promotes Endothelial Cell Proliferation in Female Photothrombotic Rats

**Figure 3:**
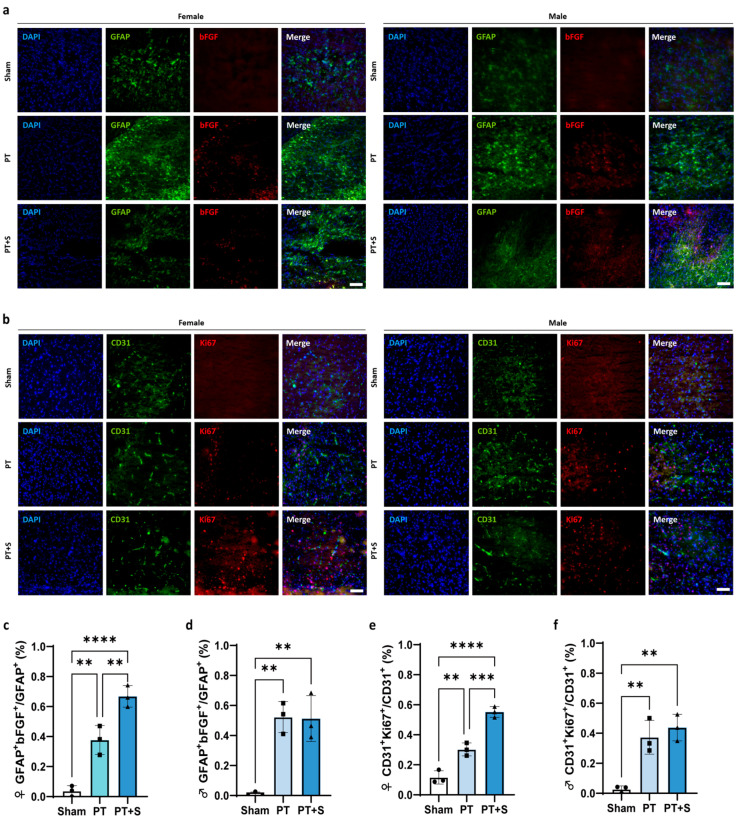
The upregulation of bFGF promotes cell proliferation in female photothrombosis rats. (**a**) Representative immunofluorescent images of glial fibrillary acidic protein (GFAP) (green) and bFGF (red) in the peri-infarct region. Bar = 100 μm. (**b**) Representative immunofluorescent images of cluster of differentiation 31 (CD31) (green) and Ki67 (red) in the peri-infarct region. Bar = 100 μm. (**c**,**d**) Quantitative analysis of bFGF expression in astrocytes: the percentage of GFAP+ bFGF+ (N = 3 rats/group) cells in the total GFAP+ cells of female rats (**c**) and male rats (**d**). (**e**,**f**) Quantitative analysis of Ki67 expression in vascular endothelial cells: the percentage of CD31+ Ki67+ (N = 3 rats/group) cells in the total CD31+ cells of female rats (**e**) and male rats (**f**). Circle: Sham group. Square: PT group. Triangle: PT + S group. All data are presented as mean ± SD. One-way ANOVA test. ** *p* < 0.01, *** *p* < 0.001, and **** *p* < 0.0001.

We next investigated whether the increased bFGF expression in female rats after laser stimulation was associated with changes in endothelial cell proliferation. Immunofluorescent staining revealed a significant increase in the proportion of GFAP+ bFGF+ cells in female rats following laser stimulation, while no difference was observed in male rats (Figure 3a,c,d). This suggests that astrocytic photogenetic stimulation upregulates bFGF specifically in female rats.

Since bFGF is known to promote endothelial cell proliferation [32,33,34], we further examined the distribution of CD31+ and Ki67+ cells in the peri-infarct region. The results showed that the number of proliferating endothelial cells (CD31+ and Ki67+) increased in the PT + S rats of the female group (Figure 3b,e,f), suggesting that bFGF upregulation after laser stimulation may promote endothelial cell proliferation in the chronic phase of stroke recovery in female rats.

### 2.4. Oxygen–Glucose Deprivation (OGD) Induced Astrocyte Activation In Vitro

**Figure 4:**
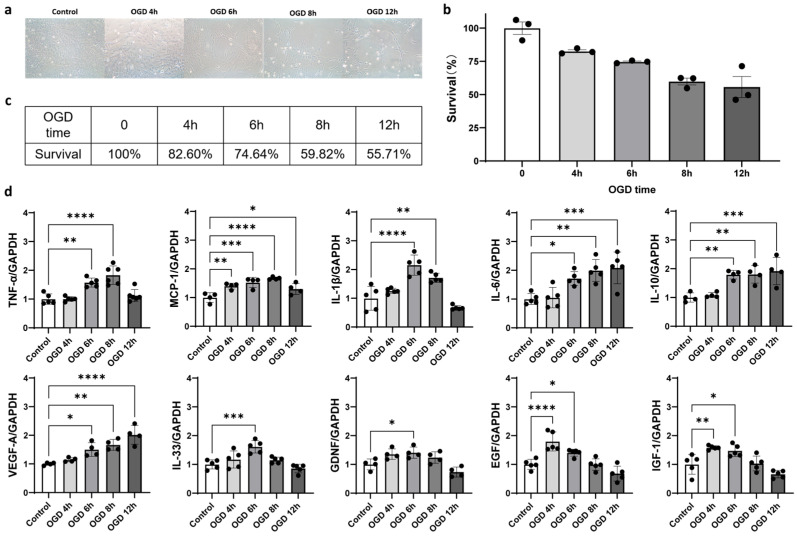
Oxygen–glucose deprivation (OGD) treatment activates astrocytes in vitro. (**a**) Bright-field images of primary astrocytes in control group and under different oxygen–glucose deprivation (OGD) treatment time (4, 6, 8, or 12 h), followed by 24 h reoxygenation. Bar = 100 μm. (**b**) CCK-8 assay to measure the cell proliferation ability of primary astrocytes in control group and under different OGD treatment time (4, 6, 8, or 12 h) plus 24 h reoxygenation. N = 3 for each group. (**c**) The cell survival rate of primary astrocytes in OGD treatment group (4, 6, 8, or 12 h) plus 24 h reoxygenation compared with control group. (**d**) RT-qPCR analysis of mRNA expression level of TNF-α, monocyte chemoattractant protein-1 (MCP-1), IL-1β, IL-6, IL-10, VEGF-A, IL-33, glial-derived neurotrophic factor (GDNF), epidermal growth factor (EGF), and insulin-like growth factor-1 (IGF-1) in control group and OGD treatment (4, 6, 8, or 12 h) groups, followed by 24 h reoxygenation. N = 4~5 for each group. All data are presented as mean ± SD. One-way ANOVA test, * *p* < 0.05, ** *p* < 0.01, *** *p* < 0.001, and **** *p* < 0.0001.

To explore the effects of bFGF on astrocyte activation [35], we used an in vitro model of oxygen–glucose deprivation (OGD), which mimics ischemic conditions. First, we optimized the OGD treatment duration to induce astrocyte activation while preserving cell viability. Bright-field images indicated that OGD treatment reduced the astrocyte viability (Figure 4a), and the cell counting kit-8 (CCK-8) assay showed a dose-dependent reduction in astrocyte survival, with approximately 70% survival after 6 h of OGD treatment (Figure 4b,c).

**Figure A2:**
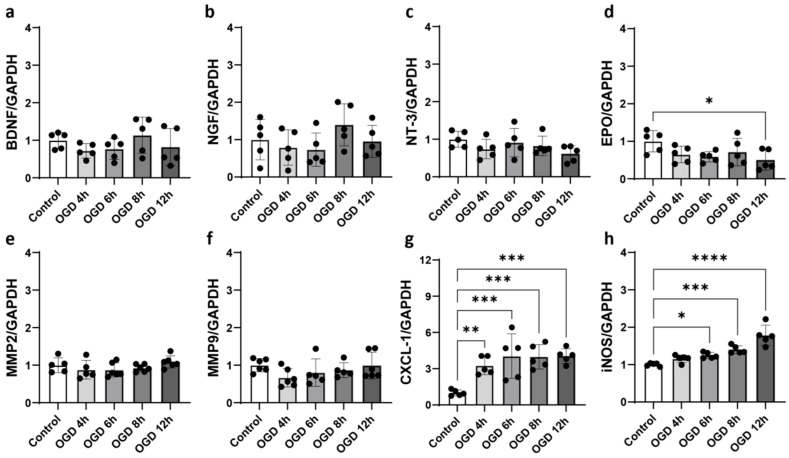
The change in other factors in astrocytes after oxygen–glucose deprivation. (**a**–**h**) RT-PCR analysis for the mRNA expression level of brain-derived neurotrophic factor (BDNF), nerve growth factor (NGF), neurotrophin-3 (NT-3), erythropoietin (EPO), MMP2, MMP9, C-X-C motif chemokine ligand 1 (CXCL-1), and iNOS in different groups. N = 5~6 for each group. PT: photothrombosis. PT + S: photothrombosis with laser stimulation. One-way ANOVA test, * *p* < 0.05, ** *p* < 0.01, *** *p* < 0.001, and **** *p* < 0.0001.

We then measured the expression of 10 neurotrophic factors and 6 inflammation factors in response to OGD treatment. Seven neurotrophic factors (VEGF-A, IL-10, IL-33, GDNF, EGF, IGF-1, and BDNF) and three inflammatory markers (TNF-α, MCP-1, and IL-1β) were significantly upregulated after 6 h of OGD treatment, confirming that this condition effectively induces astrocyte activation (Figure 4d and Figure A2a–h).

### 2.5. bFGF Inhibits Astrocyte Activation

**Figure A3:**
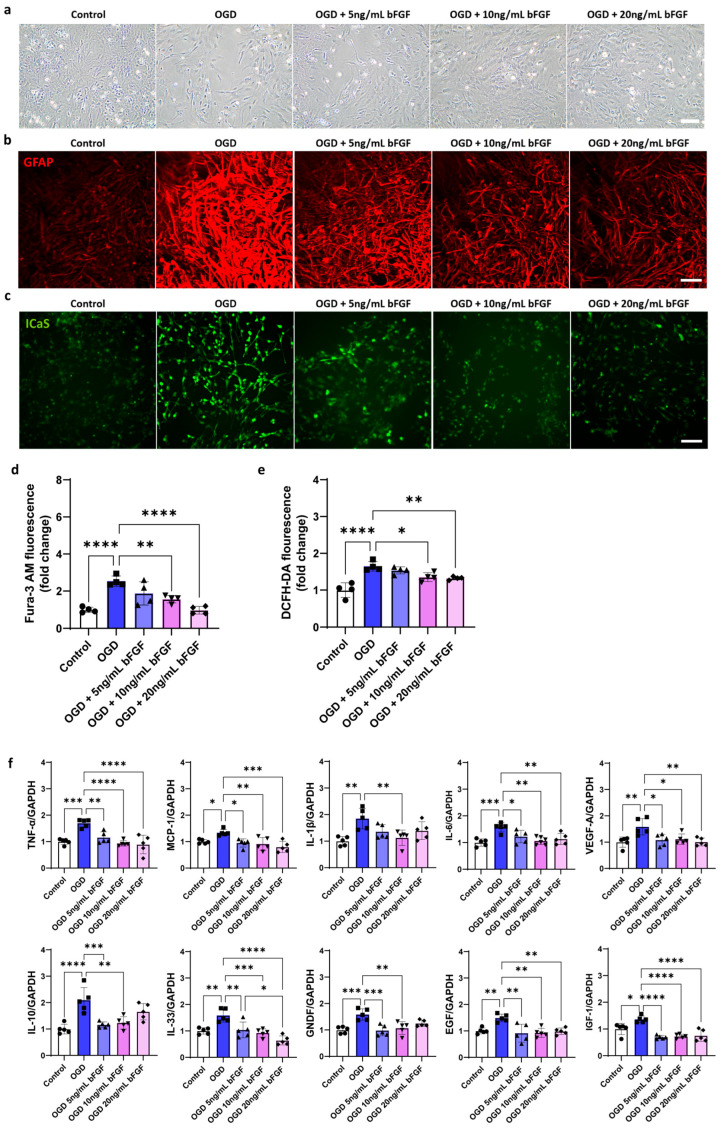
Different concentrations of bFGF have different inhibition degrees to the activation of astrocytes. (**a**) The bright-field images of primary astrocytes. Bar = 100 μm. (**b**) Representative immunofluorescent images showing the expression of GFAP (red) in primary cultured astrocytes (one of three independent experiments). Bar = 100 μm. (**c**) Intracellular calcium signal (one of four independent experiments). Bar = 100 μm. (**d**) Quantification of intracellular calcium signal in astrocytes. N = 4 for each group. (**e**) Intracellular ROS production of astrocytes. N = 4 for each group. (**f**) RT-PCR analysis for mRNA level of TNF-α, MCP-1, IL-1β, IL-6, IL-10, VEGF-A, IL-33, GDNF, EGF, and IGF-1 in astrocytes of different treatments groups in vitro. N = 5 for each group. Circle: Sham group. Square: OGD group. Regular triangle: OGD 5 ng/mL bFGF group. Inverted triangle: OGD 10 ng/mL bFGF group. Rhombus: OGD 20 ng/mL bFGF group. Control, control group. OGD, 6 h OGD treatment plus 24 h reoxygenation. OGD 5 ng/mL bFGF, 6 h OGD treatment plus 24 h reoxygenation with 5 ng/mL bFGF concentration. OGD 10 ng/mL bFGF, 6 h OGD treatment plus 24 h reoxygenation with 10 ng/mL bFGF concentration. OGD 20 ng/mL bFGF, 6 h OGD treatment plus 24 h reoxygenation with 20 ng/mL bFGF concentration. ICaS: intracellular calcium signal. All data are presented as mean ± SD. One-way ANOVA test, * *p* < 0.05, ** *p* < 0.01, *** *p* < 0.001, and **** *p* < 0.0001.

Next, we tested the effect of bFGF on astrocyte activation by applying different concentrations of bFGF (5 ng/mL, 10 ng/mL, and 20 ng/mL) to OGD-treated astrocytes. The activation of astrocytes was assessed by measuring intracellular calcium levels, reactive oxygen species (ROS), the expression of GFAP, and neurotrophic and inflammatory markers. We found that 10 ng/mL bFGF effectively reduced calcium signal, ROS production, GFAP expression, and the secretion of neurotrophic and inflammatory factors (Figure A3a–f).

**Figure 5:**
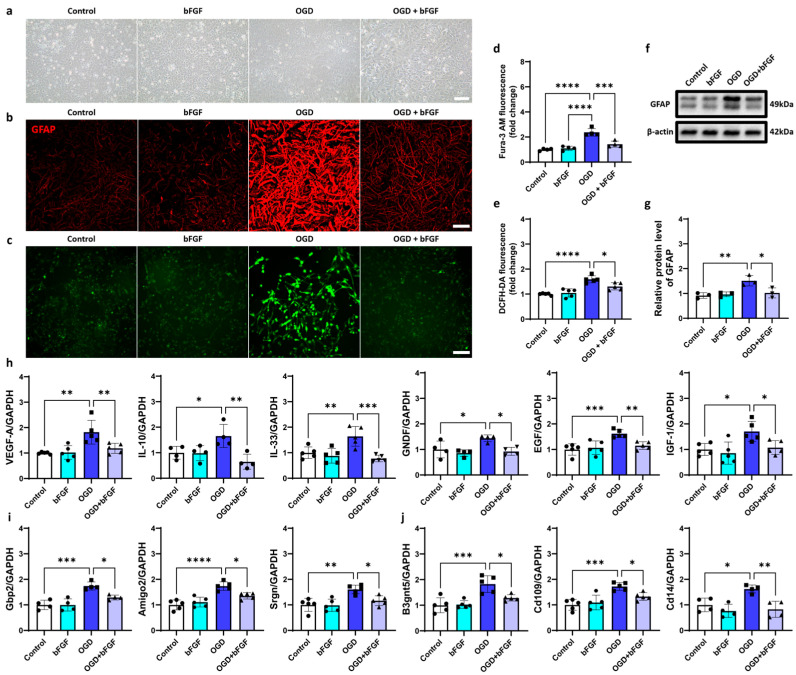
bFGF inhibits the activation of astrocytes. (**a**) Bright-field images of primary astrocytes. Bar = 100 μm. (**b**) Representative immunofluorescent images of GFAP (red) in primary astrocytes (one of three independent experiments). Bar = 100 μm. (**c**) Intracellular calcium signaling (one of four independent experiments). Bar = 100 μm. (**d**) Quantification of intracellular calcium signal in astrocytes. N = 4 for each group. (**e**) Intracellular ROS production of astrocytes. N = 5 for each group. (**f**) Western blot analysis showing the protein expression of GFAP. (**g**) Quantification of GFAP relative to β-actin and normalized to control group. N = 3 for each group. (**h**) RT-qPCR analysis for the expression levels of mRNA of VEGF-A, IL-10, IL-33, GDNF, EGF, and IGF-1 in astrocytes with different treatments in vitro. N = 4~5 for each group. (**i**) The mRNA expression level of A1-specific transcripts Gbp2, Amigo2, and Srgn in different groups. N = 4~5 for each group. (**j**) The mRNA expression level of A2-specific transcripts B3gnt5, Cd109, and Cd14 in different groups. N = 4~5 for each group. Circle: Sham group. Hexagon: bFGF group. Square: OGD group. Triangle: OGD + bFGF group. Control, control group. bFGF, cells cultured with 10 ng/mL bFGF concentration. OGD, 6 h OGD treatment followed by 24 h of reoxygenation. OGD + bFGF, 6 h OGD treatment followed by 24 h of reoxygenation with 10 ng/mL bFGF concentration. ICaS: intracellular calcium signal. All data are presented as mean ± SD. One-way ANOVA test, * *p* < 0.05, ** *p* < 0.01, *** *p* < 0.001, and **** *p* < 0.0001.

Bright-field images showed that bFGF treatment increased astrocyte survival (Figure 5a). Western blot and immunofluorescence analysis confirmed that bFGF treatment decreased the expression of GFAP, which was upregulated after OGD treatment (Figure 5b,f,g). Additionally, bFGF treatment reduced calcium overload and ROS production (Figure 5c–e). RT-qPCR results showed that bFGF downregulated the expression of several neurotrophic factors (VEGF-A, IL-10, IL-33, GDNF, EGF, and IGF-1) that are typically upregulated during astrocyte activation [36,37,38,39,40,41,42] (Figure 5i) and the expression of A1-specific transcripts (Gbp2 and Ggta1) and A2-specific transcripts (B3gnt5, Cd109, and Clcf1; Figure 5j). These results suggested that bFGF can inhibit the activation of astrocytes toward both A1 and A2 phenotypes in vitro.

## 3. Discussion

Optogenetics has become a widely used tool to modulate neuronal or glial cell activity [31,43]. Recent studies have shown that using optogenetics activation of astrocytes can aid in brain injury repair following stroke [31]. However, potential gender differences in the effectiveness of this approach remain unclear, despite growing evidence suggesting that males and females exhibit distinct pathological outcomes after stroke [44,45,46,47,48,49,50]. In this study, we treated post-stroke rats with ChR2 optogenetic activation of astrocytes and found that the male rats showed better recovery than female rats. Notably, the expression of bFGF significantly increased in female rats, but no significance was observed in male rats, after stroke and laser stimulation. Given that bFGF has been shown to inhibit astrocyte activation [25,35], this may explain why female rats exhibited poorer recovery compared to males following optogenetic stimulation.

Astrocytes become activated after brain injury and secrete various neurotrophic and inflammatory factors, which can have both beneficial and detrimental effects on recovery [51,52,53,54,55]. In this study, we induced the astrocyte activation in vitro through oxygen and glucose deprivation (OGD) and found that upregulation of both neurotrophic and inflammatory factors took place, consistent with previous research [52,56]. In addition, recent studies have proposed the concept of A1 and A2 astrocytes [10]. Both of them are activated states but exert opposing effects in neurological disorders. A1 astrocytes are induced by some inflammatory factors secreted by microglia [10]. These astrocytes have neurotoxicity and exacerbate oxidative stress in the brain [10,57]. A2 astrocytes are triggered by ischemia, upregulating trophic factors to aid repair [10]. In this study and our previous study, optogenetic activation of astrocytes demonstrated beneficial effects in post-stroke rats, and no significant upregulation of inflammatory factors was observed [31], indicating that optogenetic stimulation may preferentially enhance the A2 phenotype, aligning with existing research [58]. In vitro, OGD-induced activation concurrently increased the ROS level and upregulated the expression of inflammatory markers and neurotrophic factors, implying that OGD may drive astrocytes toward mixed A1/A2 states. In this study, we also confirmed the above hypothesis by testing the expression of A1-specific transcripts and A2-specific transcripts under optogenetic stimulation and OGD situation.

Interestingly, during OGD-induced astrocyte activation in our experiments in vitro, we observed a biphasic expression pattern in certain factors, an initial upregulation followed by subsequent downregulation. We propose that this phenomenon may reflect the dynamic balance between cellular stress responses and viability. During shorter OGD durations where cell viability remains high, astrocytes actively upregulate both inflammatory and neurotrophic factors as compensatory mechanisms. Meanwhile, when OGD persistence surpassed cellular compensatory capacity, significant cell dysfunction occurs, leading to suppression of transcriptional factors. This explains the subsequent decline in factor expression. Moreover, these dynamic changes in inflammatory factor expression patterns reveal functional interrelationships among the mediators. For example, under short-term OGD (0~6 h), IL-6 expression was progressively induced. Since IL-6 enhances the expression of TNF-α and IL-1β [59], all three cytokines showed significant upregulation compared to the control group after 6 h OGD treatment. Additionally, the expression of IL-10 showed a significant increase compared to the control group when OGD treatment reached 6 h. Given IL-10’s inhibitory effects on IL-33 expression [60], the expression level of IL-33 was decreased after 6 h of OGD treatment. These phenomena reflect that astrocytes have different temporal expression profiles in inflammatory environments.

bFGF is a multifunctional cytokine involved in neural development, angiogenesis, and healing of brain injuries [34,61,62,63,64,65]. Both exogenous and endogenous bFGF have similar effects in the nervous system [63], where they exert an inhibitory effect on astrocyte activation [25,35]. This effect occurs mainly through the TLR4/NFκB signaling pathway, leading to reduced GFAP and IL-6 expression [25]. In this experiment, astrocytes were activated via OGD treatment, and subsequent bFGF treatment led to a decrease in GFAP, which supports the previously reported inhibitory role of bFGF in astrocyte activation [18,30]. Notably, bFGF treatment also reduced ROS, inflammatory markers, and neurotrophic factors, indicating a suppressive effect on both A1 and A2 phenotypes. This suggests that bFGF’s inhibitory effect on A2 astrocyte activation could contribute to the poorer recovery observed in female rats after optogenetic activation.

Our findings indicate that the higher expression of bFGF in female rats following stroke and optogenetic activation inhibits astrocyte activation, leading to downregulation of neurotrophic factors and poorer recovery outcomes. Although bFGF treatment reduced levels of reactive oxygen species and inflammatory factors in astrocytes—likely beneficial to recovery—its inhibition of neurotrophic factors may outweigh these effects. This could help explain the poorer recovery in female rats. Therefore, our data suggest that bFGF mediates a reduction in neurotrophic factors that inhibit astrocyte activation following optogenetic stimulation.

This study primarily focused on the third day after stroke for sample collection and analysis, thus limiting its temporal scope. The long-term impact of bFGF on the recovery after stroke in rats after optogenetic activation of astrocytes is still unclear. bFGF is known to promote angiogenesis, which can improve cerebral perfusion, enhance neuronal survival, and facilitate brain plasticity and neurological recovery post-stroke [32,33,34,66,67,68,69]. However, the repair effect of angiogenesis is often delayed and may only become apparent in the subacute or chronic phases of recovery [66,69,70,71]. This could explain why the endothelial cell proliferation was observed in our experiment but did not lead to more significant recovery in female rats. Future studies with a longer time scale will be necessary to confirm this hypothesis.

In conclusion, our study demonstrates that there are gender differences in the repair of post-stroke brain injury through optogenetic astrocyte activation. Specifically, we found that the increased expression of bFGF in female rats after stroke and optogenetic stimulation contributes to these differences. Additionally, we confirmed the inhibitory effect of bFGF on astrocyte activation in vitro. As a result, neurofunctional recovery in female rats differs from that in males during the acute phase of stroke. This research provides important evidence for the role of astrocytes in gender-specific recovery during the acute stage of ischemic stroke.

## 4. Materials and Methods

### 4.1. Animal Experimental Design

Animal experiments were conducted in accordance with the Animal Research: Reporting of In Vivo Experiments (ARRIVE) guidelines and received approval from the Institutional Animal Care and Use Committee of Shanghai Jiao Tong University, Shanghai, China.

### 4.2. Reagents

Basic fibroblast growth factor (bFGF) was purchased from Novoprotein (Suzhou, China) and was dissolved in double distilled water to prepare a 10 μg/mL solution.

### 4.3. Photothrombotic (PT) Ischemic Model

Sprague-Dawley rats, aged 8~10 weeks, were anesthetized with 1.5–2% isoflurane in the mixed gas of 30% O_2_/68.5–69% NO. Then, the rat’s head was fixed by a stereotactic frame device, and anesthesia was maintained during the operation using a mask. A midline vertical incision was made from the interocular region to the cervical area, followed by scalp and periosteum removal to expose the skull. After identifying the bregma, a point 3 mm lateral to the left was marked as the center of a 1.5 mm radius circular craniotomy site. A blunt skull drill was used to thin the circle area covered on the skull. Then, Rose Bengal (0.05 mg/g, Sigma Aldrich, St. Louis, MO, USA) was injected through the tail vein. After covering the other parts with black tape with a diameter of 3 mm to limit the laser-stimulated area, the stimulation of a green laser (532 nm green diode-pumped solid state (DPSS) laser, 80 mW, Shanghai Laser & Optics Century Co., Ltd., Shanghai, China) was applied for 15 min. Then, a hole was drilled near the infarcted area of the sensory motor cortex using a pointed skull drill to insert a ceramic fiber (200 μm optical fiber; both sides are penetrated; the length of the exit section is 2.5 mm; Ximu Instrument Co., Ltd., Shanghai, China) to implement laser stimulation. After surgery, three skull nails (cross-thread, 3 mm in length and 1.0 mm in diameter) and denture base resin (Zhangjiang Biomaterials Co., Ltd., Shanghai, China) were used to fix the fiber.

### 4.4. Laser Stimulation

Astrocytes of GFAP-ChR2-EYFP transgenic rats were used for the stimulation group. We used a 473 nm pulsed blue laser (473 nm Blue DPSS Laser, 20 Hz, 0.55 mW, Shanghai Laser & Optics Century Co., Ltd. (SLOC), BL473T3-050FC, Shanghai, China) to perform laser stimulation at 24, 36, 48, and 60 h after the photothrombotic ischemic model [31,72].

### 4.5. Neurobehavioral Function Examination

Neurobehavioral function examination was performed using the modified neurologic severity score (mNSS) [73,74]. This comprehensive scoring system evaluates the extent of neurological impairment by considering the motor, reflex, and sensory capabilities of the experimental animals. On days 1 and 3 in the PT group rats, another investigator, who was blinded to the experimental conditions, applied the mNSS, which ranges from 0 to 14 points. The score between 1 and 4 indicates mild injury, the score from 5 to 9 indicates moderate injury, and the score from 10 to 14 indicates severe injury. The normal score is 0, while the maximum injury score is 14.

### 4.6. Cell Culture

Primary astrocytes were prepared from cerebral cortices of 1-day-old neonatal Sprague-Dawley rats. The cerebral cortices were isolated from the brain and then digested with 0.25% trypsin at 37 °C for 3 min. The digestion was stopped with Dulbecco’s Modified Eagle Medium (DMEM; MA0212, Meilunbio, Dalian, China) supplemented with 10% fetal bovine serum (FBS; 16141-079, Gibco, New York, NY, USA), 100 units/mL penicillin, and 100 μg/mL streptomycin. Then, the cells were filtered through a 70 μm cell strainer. After centrifuging and resuspending, the isolated cells were cultured in Dulbecco’s Modified Eagle Medium for 7 d, until they reached 80%~90% confluence. The purity of the astrocytes was confirmed by immunofluorescence staining for GFAP.

### 4.7. Oxygen–Glucose Deprivation and Reoxygenation Treatment in Cell Model

Following the cell culture procedure listed in Section 4.6, primary astrocytes were washed twice with PBS and the culture medium was replaced with glucose free DMEM (MA0598, Meilunbio, Dalian, China). Then the culture plates with primary astrocytes were placed into a sealed chamber with 1% O_2_ at 37 °C for 4, 6, 8, or 12 h.

For reoxygenation, the culture plates with primary astrocytes were retrieved from the sealed chamber. Then, primary astrocytes were washed twice with PBS, the glucose-free medium was replaced with initial culture medium, and the cells were moved to the incubator for 24 h.

### 4.8. Brain Infarct and Edema Volume Measurements

On the third day after PT treatment, all adult rats from each group were euthanized and perfused with PBS. The brains were rapidly frozen in isopentane (−80 °C, 78-78-4, Macklin, Shanghai, China) for 1 min and then stored at −80 °C for 1 d. Coronal brain sections, 20 μm thick, were cut using a microtome and mounted on slides from the onset to the end of the infarct. Infarct and edema volumes were measured using cresyl violet staining. The infarct volume was calculated as the total volume of the contralateral hemisphere minus the volume of the normal region in the ipsilateral hemisphere. The edema volume was determined as the total volume of the ipsilateral hemisphere minus the volume of the contralateral hemisphere. The infarct or edema volume was calculated using the following formula:

(1) $$V=\sum \frac{h}{3}\left[{\Delta S}_{n}+{\left({\Delta S}_{n}\times {\Delta S}_{n+1}\right)}^{\frac{1}{2}}+{\Delta S}_{n+1}\right]$$

where ΔS_n_ and ΔS_n+1_ represent the infarct or edema area of two adjacent sections, and h denotes the distance between two adjacent sections (h = 300 μm).

### 4.9. Quantitative Real-Time PCR Assessment

Total RNA of the ipsilateral cortex was collected and extracted using TRIzol reagent (R411-01, Vazyme, Nanjing, China). RNA concentration was measured, and RNA quality was determined via A260/280, using a spectrophotometer (NanoDrop1000, Thermo Fisher, Wilmington, NC, USA). RNA samples were reverse-transcribed to cDNA with an RT Kit (13894ES60, Yeasen, Shanghai, China). Then, quantitative real-time PCR was performed using cDNA, with a SYBR Green Kit (11143ES80, Yeasen, Shanghai, China), under the following cycling conditions: 95 °C for 5 min, followed by 40 cycles of 95 °C for 10 s and 60 °C for 30 s. The 2^−△△Ct^ method was used for relative quantification of the target gene.

The primer sequences of the target genes are listed in Table 1.

### 4.10. Western Blot Analysis Determination

Brain tissues or primary cultured astrocytes were dissolved in extraction buffer. WE used a 1 mL extraction buffer that was composed of 100 μL 10 × PhosSTOP (04906837001, Roche, Basel, Switzerland), 10 μL 100 × cOmplete Tablets (04693132001, Roche, Basel, Switzerland), 10 μL 100 × PMSF (XW020003, Sinopharm, Shanghai, China), 100 μL 10 × RIPA (20-188, Merek, Kenilworth, NJ, USA), and 780 μL ddH_2_O. Equal amounts of proteins were electrophoresed on SDS-PAGE (12.5%, Epizyme, Shanghai, China) and then transferred to a PVDF membrane (0.45 mm, RIPA, Millipore, Burlington, MA, USA). The membrane was blocked at room temperature for 15 min with protein-free rapid blocking buffer (PS108P, Epizyme, Shanghai, China) and then incubated with the primary antibodies (1:1000) at 4 °C for 16 h. The primary antibodies include bFGF antibody (05-118, Millipore, Boston, MA, USA), GFAP (ab53554, abcam, Shanghai, China), and β-actin (66009, proteintech, Rosemont, IL, USA) at 4 °C overnight. After washing with 1 × TBST buffer (60145ES76, Yeasen, Shanghai, China), all the membranes were incubated for 1 h at room temperature with horseradish peroxidase (HRP)-conjugated secondary antibodies (1:5000, anti-mouse or anti-goat IgG, Huabio, Hangzhou, China). The bands were detected using enhanced chemiluminescence substrate (Meilunbio, Shanghai, China) and quantized by Image J 1.8.0 software (NIH, Bethesda, MD, USA).

### 4.11. Immunofluorescence Staining

The brain sections were fixed with 4% PFA for 10 min and 0.3% Triton X-100 solution for 10 min. All sections were blocked with 1% bovine serum albumin (BSA, GIBCO, Waltham, MA, USA) for 1 h and then incubated with rabbit-anti bFGF (1:100; HUABIO, Hangzhou, China), goat-anti GFAP (1:200; Abcam, Shanghai, China), rabbit-anti Ki67 (1:200; MCE, Shanghai, China), goat-anti CD31 (1:200; R&D, Minneapolis, MN, USA), and rabbit-anti GFAP (1:200; Millipore, Boston, MA, USA) overnight, at 4 °C, under humidified conditions. After washing with PBS, the sections were incubated with the corresponding fluorescent-conjugated secondary antibodies for 1 h at 37 °C and observed under a Scientific CMOS microscope (K-8; Leica, Wetzlar, Germany). The following secondary antibodies were used: Alexa Fluor 594-conjugated donkey anti-rabbit secondary antibody (1:400; Invitrogen, Waltham, MA, USA), and Alexa Fluor 488-conjugated donkey anti-goat secondary antibody (1:400; Invitrogen, Waltham, MA, USA). The genuine target staining was distinguished from the background with a secondary antibody only.

### 4.12. Reactive Oxygen Species Production Assay

Reactive oxygen species (ROS) production assay was performed as described in previous research [75]. Primary astrocytes were seeded in a black 96-well cell culture plate loaded with 10 μM DCFH-DA (MA0219, MeilunBio, Dalian, China) at 37 °C for 30 min. Cells were then washed with Dulbecco’s Modified Eagle Medium (DMEM; MA0212, Meilunbio, Dalian, China) three times and then scanned by a microplate reader.

### 4.13. Intracellular Calcium Measurement

Intracellular calcium measurement was performed as described in previous research [76]. Primary astrocytes were seeded on bacteria-free cover glass in 24-well cell culture plate loaded with 100 μL 2 μM Fura-3 AM (HY-D0716, MedChemExpress, Monmouth Junction, NJ, USA) at 37 °C for 30 min. Cells were then washed with Dulbecco’s Modified Eagle Medium (DMEM; MA0212, Meilunbio, Dalian, China) three times for 5 min each time, and the signal was observed with fluorescence microscope.

### 4.14. Statistical Analysis

All data were from at least three individual biological replicates and were displayed as mean ± SD. Unpaired two-tailed t-test was used for variables with normal distribution to evaluate the difference between two independent groups. Mann–Whitney U test was employed to assess differences between two independent groups for non-normally distributed variables. The significance of differences beyond two groups was analyzed by one-way ANOVA. When the *p*-value is less than 0.05, the difference is considered statistically significant. GraphPad 9.0 software was used for the analysis.

## Figures and Tables

**Table 1:** RT-qPCR primer sequences.

Name	Forward Primer (5′–3′)	Reverse Primer (5′–3′)
bFGF	CAACACTTACCGGTCACGGA	CCCCGTTTTGGATCCGAGTT
IL-10	GCTCTTACTGGCTGGAGTGAG	CTCAGCTCTCGGAGCATGTG
IL-6	TCCTACCCCAACTTCCAATGCTC	TTGGATGGTCTTGGTCCTTAGCC
IL-1β	CACCTCTCAAGCAGAGCACAG	GGGTTCCATGGTGAAGTCAAC
TNFα	AAATGGGCTCCCTCTCATCAGTTC	TCTGCTTGGTGGTTTGCTACGAC
TGFβ	CCACCTCCTGAGGGTGCTT	ATGTCGGAGAAAGGCGCG
IFNγ	GTCATCGAATCGCACCTGA	GTGCTGGATCTGTGGGTTG
iNOS	TTYGATGTGCTGCCTCTGGT	CATTCTGCTTCTGGAAACTATGG
MMP2	CTGATAACCTGGATGCAGTCGT	CCAGCCAGTCCGATTTGA
MMP9	ATGTCACTTTCCCTTCACCT	TTAGAGCCACGACCATACAG
VEGF-A	GAGTCTGTGCTCTGGGATTTG	TCCTGCTACCTCTTTCCTCTG
IL-33	CCCTGAGCACATACAACGACC	CACCATCAGCTTCTTCCCATC
GDNF	ATTCCTGGCGTTACCTTG	CTGTATTCCGTCTCCTTGG
BDNF	CTACGAGACCAAGTGTAATC	TTATGAATCGCCAGCCAAT
NGF	GAGCGCATCGCTCTCCTT	GAGCGCATCGCTCTCCTT
NT-3	GGTGAACAAGGTGATGTCCATC	GGCAGGGTGCTCTGGTAATTTTCCT
EPO	TCCTTGCTACTGATTCCTCTCTGG	AAGTATCCGCTGTGAGTGTTCG
EGF	CTTAGGGATGTGGGGGACTT	TTGGGCTGTTGGTGTTCCTC
IGF-1	CAGTTCGTGTGTGGACCAAG	TCAGCGGAGCACAGTACATC
CXCL	TGAGCTGCGCTGTCAGTGCCT	AGAAGCCAGCGTTCACCAGA
MCP	GCGCCGGAAAGCTGTAGATG	TTTGCTTGTCCAGGTGGTC
IL-1α	TCAAAGATGTCCACCTTCACC	CTGATCTGGGTTGGATGGTC
C1q	TTCTGTGACTATGCCTACAACAC	GCCCAGTAGTGAGTTCTTGTC
C3	GCCAGCAGCTCTACAATGTG	GACTGCCACTTTCCCATAGC
Amigo2	GTTCGCCACAACAACATCAC	GTTTCTGCAAGTGGGAGAGC
Gbp2	TAAAGGTCCGAGGCCCAAAC	AACATATGTGGCTGGGCGAA
Ggta1	TCTCAGGATCTGGGAGTTGGA	GAGTTCTATGGAGCTCCCGC
Srgn	GTTCAAGGTTATCCTGCTCGGA	AAACAGGATCGGTCATCGGG
B3gnt5	TGCTCCTGGATGAAAGGTCC	ACATGCTTGATCCGTGTGGT
Cd14	TCAGAATCTACCGACCATGAAGC	GGACACTTTCCTCGTCCTGG
Cd109	GTCGCTCACAGGTACCTCAA	CTGTGAAGTTGAGCGTTGGC
Clcf1	GACTCGTGGGGGATGTTAGC	CCCCAGGTAGTTCAGGTAGGT
Emp1	ACCATTGCCAACGTCTGGAT	TGGAACACGAAGACCACGAG
GAPDH	GATGGTGAAGGTCGGTGTGA	TGAACTTGCCGTGGGTAGAG

## Data Availability

Data are contained within the article.

## Abbreviations

The following abbreviations are used in this manuscript:

Amigo2	adhesion molecule with Ig-like domain 2
B3gnt5	β-1,3-N-acetylglucosaminyltransferase 5
BBB	blood–brain barrier
BDNF	brain-derived neurotrophic factor
bFGF	basic fibroblast growth factor
C1q	complement component 1q
C3	complement component 3
CCK-8	cell counting kit-8
Cd14	cluster of differentiation 14
Cd31	cluster of differentiation 31
Cd109	cluster of differentiation 109
ChR2	channelrhodopsin 2
Clcf1	cardiotrophin-like cytokine factor 1
CXCL	C-X-C motif chemokine ligand
EGF	epidermal growth factor
Emp1	epithelial membrane protein 1
EPO	Erythropoietin
EYFP	enhanced yellow fluorescent protein
Gbp2	guanine-binding protein 2
GDNF	glial cell line-derived neurotrophic factor
GFAP	glial fibrillary acidic protein
Ggta1	α-1,3-galactosyltransferase
IFNγ	interferon γ
IGF-1	insulin-like growth factor-1
IL-1α	interleukin-1α
IL-1β	interleukin-1β
IL-6	interleukin-6
IL-10	interleukin-10
IL-33	interleukin-33
iNOS	inducible nitric oxide synthase
MCP	monocyte chemotactic protein
MMP2	matrix metalloproteinase 2
MMP9	matrix metalloproteinase 9
mNSS	modified neurologic severity score
NGF	nerve growth factor
NT-3	neurotrophin-3
OGD	oxygen–glucose deprivation
PT	photothrombotic
PT + S	photothrombotic stroke group with laser stimulation
ROS	reactive oxygen species
Srgn	serglycin
TGFβ	transforming growth factor-β
TNF-α	tumor necrosis factor-α
VEGF	vascular endothelial growth factor

## Appendix A

**Table A1:** The number of rats corresponding to each analysis.

Figure	Group and Number
Figure 1b	PT (N = 10); PT + S (N = 16)
Figure 1c	PT (N = 7); PT + S (N = 13)
Figure 1f,h	N = 5 for each group
Figure 1g,i	N = 5 for each group
Figure 2a	Sham (N = 3~6); PT (N = 6~9); PT + S (N = 6~7)
Figure 2c	N = 3 for each group
Figure 2d	Sham (N = 5); PT (N = 8); PT + S (N = 6~7)
Figure 2e	Sham (N = 5); PT (N = 7~8); PT + S (N = 7)
Figure 2f	Sham (N = 4~5); PT (N = 6~8); PT + S (N = 6~7)
Figure 3c	N = 3 for each group
Figure 3d	N = 3 for each group
Figure 3e	N = 3 for each group
Figure 3f	N = 3 for each group
Figure 4b	N = 3 for each group
Figure 4d	N = 4~5 for each group
Figure 5d	N = 4 for each group
Figure 5e	N = 5 for each group
Figure 5g	N = 3 for each group
Figure 5h	N = 4~5 for each group
Figure 5i	N = 4~5 for each group
Figure 5j	N = 4~5 for each group
Figure A2a–h	N = 5~6 for each group
Figure A3d	N = 4 for each group
Figure A33e	N = 4 for each group
Figure A3f	N = 5 for each group
